# Chiral emission and Purcell enhancement in a hybrid plasmonic-photonic microresonator

**DOI:** 10.1038/s41377-019-0241-z

**Published:** 2020-01-06

**Authors:** Qi-Tao Cao, You-Ling Chen, Yun-Feng Xiao

**Affiliations:** 1 Frontiers Science Center for Nano-optoelectronics & School of Physics, Peking University, Beijing, 100871 China; 2 State Key Laboratory on Integrated Optoelectronics, Institute of Semiconductors, Chinese Academy of Sciences, Beijing, 100083 China

**Keywords:** Microresonators, Nanophotonics and plasmonics

## Abstract

A high-Q hybrid plasmonic-photonic microresonator, which consists of a dielectric microdisk hybridized with a plasmonic nanoantenna dimer, enables an enlarged local density of states of the optical field and chiral propagation of photons inside the cavity.

The light–matter interaction lies at the heart of both fundamental theoretical research and advanced applications in modern physics. The interaction strength typically hinges on the scale of the fine structure constant *α* ≈ 1/137, indicating an ultraweak coupling strength between light and matter in vacuum. Due to the well-known Fermi’s golden rule and Purcell effect^1^, the local density of states (LDOS) of the optical field can be engineered by a resonator, enabling an enhancement or control of the spontaneous emission rate of a quantum emitter. Over the past decades, much progress has been made using various micro/nanoresonators to enhance the light–matter interaction, especially for a Purcell enhancement.

Among the diverse candidates, optical dielectric microcavities, and localized surface plasmon resonances (LSPRs) have been studied extensively to tailor the coupling between quantum emitters and photons^2,3^. Optical dielectric microcavities feature ultrahigh *Q* factors, which correspond to very long lifetimes of the photons inside the cavity, while LSPRs have extremely small mode volumes that enable an ultrastrong coupling strength. Recently, by incorporating the merits of both optical microcavities and LSPRs, schemes of hybrid plasmonic-photonic resonators have been proposed to further promote the interplay between quantum emitters and photons^4–7^. In such a hybrid system, the optical microcavity is used to engineer the electromagnetic environment, and the LSPR usually acts as an amplifier of the optical field.

In previous hybrid strategies, whispering-gallery-mode (WGM) or Fabry-Pérot-type microcavities have been widely employed for the photonic resonance, and the LSPR has been generated by a single metallic nanoparticle or nanowire. In addition to the elementary hybrid plasmonic-photonic scheme, which involves a single plasmonic nanoantenna, multiple metallic nanoparticles with a particular phase relationship can provide a new degree of freedom to tailor the light-emitter interaction. In a recent publication, Cognée et al. theoretically proposed and experimentally explored a high-Q hybrid plasmonic-photonic resonator consisting of a dielectric WGM microdisk and a plasmonic antenna dimer (Fig. 1). An enlarged-LDOS enhancement of the optical field and chiral emission inside the cavity were obtained^8^.

**Fig. 1:**
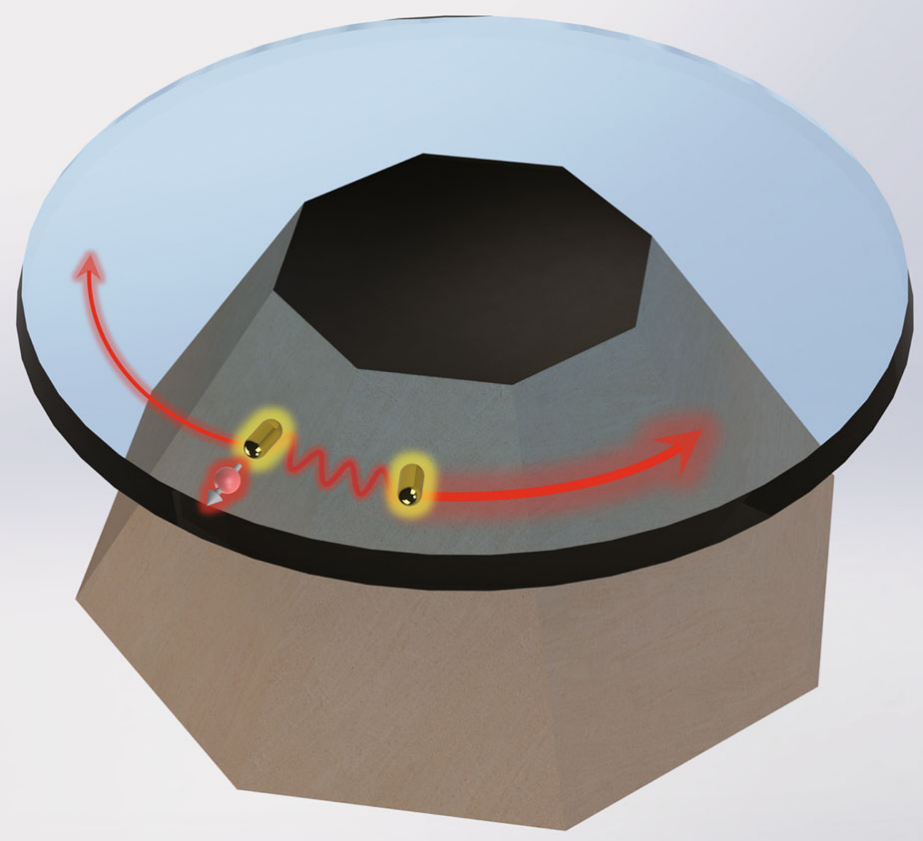
Schematic illustration of the physical model in the work^8^, where a hybrid plasmonic-photonic system consisting of a silicon nitride microdisk and a pair of radially oriented aluminum nanorods. One nanorod is driven by a dipolar emitter, resulting in chiral emission of photons in the cavity and a remarkable enhancement of the LDOS.

By introducing the notion of phased-array antenna physics into the hybrid system, the researchers investigated the influence of the antenna separation on the perturbed mode frequencies and *Q* factors. When a dipole emitter interacts with the hybrid resonator, it is found that the Purcell effect can be regulated by controlling the distance, or the phase difference, between the pair of plasmonic antennas. A maximum LDOS enhancement of almost 700 is obtained according to an analytical calculation and a full-wave simulation, benefiting from the cooperative scattering engineered by the dipole–dipole coupling in the resonator. Furthermore, different from the case of a single antenna, the plasmonic antenna dimer can induce asymmetric scattering of the optical field, resulting in the chiral propagation of photons inside the cavity under a particular phase condition and a large Purcell enhancement at the same time. Although similar chiral behavior has been reported by utilizing dielectric scatterers on WGM cavities^9^, the present work is the first plasmonic implementation. The mechanism is distinguishable by tuning the phase relationship of the plasmonic dimer rather than the mode coalescence.

This work sheds light on a new degree of freedom that can be used to tailor the interaction between light and matter using a hybrid plasmonic-photonic resonator decorated with a metallic nanoantenna dimer. The chiral propagation of photons has been realized. Moreover, from a reciprocal perspective, a selective excitation of the quantum emitter may also be achieved by controlling the phase and amplitude of the external input. Such a realization of chirality by hybridization could further be developed for chiral quantum optics, which offers fundamentally new functionalities and applications based on a propagation-direction-dependent interaction^10^. In addition, triggered by this strategy, many scenarios that contain rich physics could be explored in the future, such as deep strong coupling^11^, non-Hermitian physics^12^, and nonlinear optical effects^13^.

## References

[1] Purcell EM (1946). Spontaneous emission probabilities at radio frequencies. Phys. Rev..

[2] Vahala KJ (2003). Optical microcavities. Nature.

[3] Xu D (2018). Quantum plasmonics: new opportunity in fundamental and applied photonics. Adv. Opt. Photonics.

[4] Xiao YF (2012). Strongly enhanced light-matter interaction in a hybrid photonic-plasmonic resonator. Phys. Rev. A.

[5] de Leon NP (2012). Tailoring light-matter interaction with a nanoscale plasmon resonator. Phys. Rev. Lett..

[6] Yin Y (2016). Localized surface plasmons selectively coupled to resonant light in tubular microcavities. Phys. Rev. Lett..

[7] Peng P (2017). Enhancing coherent light-matter interactions through microcavity-engineered plasmonic resonances. Phys. Rev. Lett..

[8] Cognée KG (2019). Cooperative interactions between nano-antennas in a high-Q cavity for unidirectional light sources. Light.: Sci. Appl..

[9] Peng B (2016). Chiral modes and directional lasing at exceptional points. Proc. Natl Acad. Sci. USA.

[10] Lodahl P (2017). Chiral quantum optics. Nature.

[11] Kockum AF (2019). Ultrastrong coupling between light and matter. Nat. Rev. Phys..

[12] Miri MA, Alù A (2019). Exceptional points in optics and photonics. Science.

[13] Strekalov DV (2016). Nonlinear and quantum optics with whispering gallery resonators. J. Opt..

